# Analyzing structural alterations of mitochondrial intermembrane space superoxide scavengers cytochrome-c and SOD1 after methylglyoxal treatment

**DOI:** 10.1371/journal.pone.0232408

**Published:** 2020-04-30

**Authors:** Hilda Mercado-Uribe, Mariana Andrade-Medina, Juan Horacio Espinoza-Rodríguez, Mauricio Carrillo-Tripp, Christian Quintus Scheckhuber

**Affiliations:** 1 Centro de Investigación y de Estudios Avanzados, del Instituto Politécnico Nacional Unidad Monterrey, Parque PIIT, Apodaca, Nuevo León, México; 2 Universidad de las Américas Puebla, Escuela de Ingeniería, San Andrés Cholula, Puebla, México; School of Pharmacy, Ardabil University of Medical Sciences, ISLAMIC REPUBLIC OF IRAN

## Abstract

Mitochondria are quantitatively the most important sources of reactive oxygen species (ROS) which are formed as by-products during cellular respiration. ROS generation occurs when single electrons are transferred to molecular oxygen. This leads to a number of different ROS types, among them superoxide. Although most studies focus on ROS generation in the mitochondrial matrix, the intermembrane space (IMS) is also important in this regard. The main scavengers for the detoxification of superoxide in the IMS are Cu, Zn superoxide dismutase (SOD1) and cytochrome-c. Similar to ROS, certain reactive carbonyl species are known for their high reactivity. The consequences are deleterious modifications to essential components compromising cellular functions and contributing to the etiology of severe pathological conditions like cancer, diabetes and neurodegeneration. In this study, we investigated the susceptibility of SOD1 and cytochrome-c to *in vitro* glycation by the dicarbonyl methylglyoxal (MGO) and the resulting effects on their structure. We utilized experimental techniques like immunodetection of the MGO-mediated modification 5-hydro-5-methylimidazolone, differential scanning calorimetry, fluorescence emission and circular dichroism measurements. We found that glycation of cytochrome-c leads to monomer aggregation, an altered secondary structure (increase in alpha helical content) and slightly more compact folding. In addition to structural changes, glycated cytochrome-c displays an altered thermal unfolding behavior. Subjecting SOD1 to MGO does not influence its secondary structure. However, similar to cytochrome-c, subunit aggregation is observed under denaturating conditions. Furthermore, the appearance of a second peak in the calorimetry diagram indirectly suggests de-metallation of SOD1 when high MGO levels are used. In conclusion, our data demonstrate that MGO has the potential to alter several structural parameters in important proteins of energy metabolism (cytochrome-c) and antioxidant defense (cytochrome-c, SOD1).

## Introduction

Glycation, in contrast to glycosylation, is a non-enzymatic reaction of amino groups in biomolecules with sugars and sugar-derived molecules, for example dicarbonyls like methylglyoxal (MGO) [1].

The glycolysis intermediates dihydroxyacetone phosphate (DHAP) and glyceraldehyde 3-phosphate can give rise to MGO by phosphate elimination [2]. This process occurs spontaneously. L-threonine catabolism is a further possible route for MGO generation via aminoacetone oxidation. The enzyme semicarbazide-sensitive amine oxidase is responsible for this reaction [3]. It should be noted that the fragmentation of lipid peroxides can also lead to the formation of various reactive carbonyl species, among them MGO and glyoxal [4]. In cultured cells and tissues MGO levels are typically between 0.3–6 μM [5], however, high concentrations of up to 310 μM were described in healthy Chinese hamster ovary cells [6].

**Fig 1:**
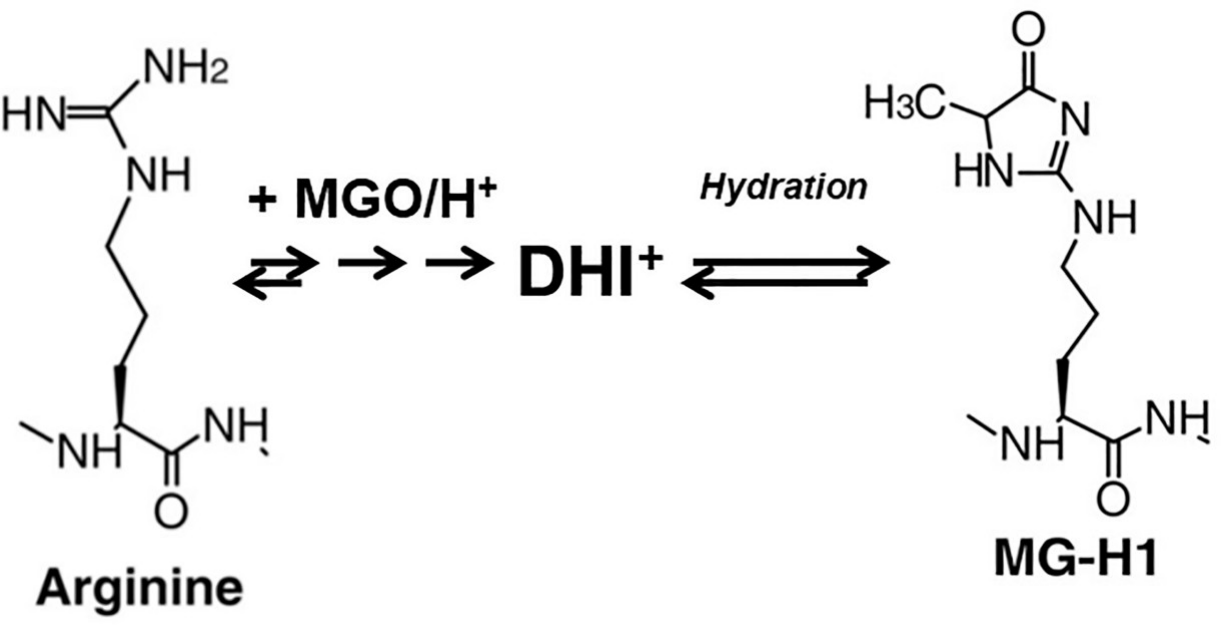
Glycation reaction of arginine with MGO. MGO preferably reacts with the amino acid arginine. As an irreversible intermediate, the AGE dihydroxyimidazoline (DHI) is formed after Schiff base addition and its subsequent rearrangement (Amadori product formation). DHI can be protonated to yield a cation. Hydration of DHI leads to the formation of the AGE 5-hydro-5-methylimidazolone (MG-H1). This is a reversible reaction.

Regarding dicarbonyl reaction chemistry, a Schiff base is initially formed between the amine and the aldehyde group of the reacting molecules. The Schiff base is subsequently re-arranged to form an Amadori product. Further reactions involving the Amadori product ultimately lead to the generation of advanced-glycation end products (AGEs) (Fig 1) [7, 8]. AGE formation occurs mostly in proteins but it is also observed in nucleic acids and lipids [9]. This modification is usually considered detrimental as structure and function of the target are often compromised. Elevated levels of dicarbonyls and AGEs are indicative of numerous severe disease conditions, among them neurological disorders, cancer and diabetic nephropathy [10, 11]. In order to maintain low levels of glycating compounds cells harbor specific defense systems. The most prominent is the glyoxalase system that comprises glyoxalase I (GLO1), glyoxalase II (GLO2) and catalytic amounts of reduced glutathione (GSH) [12, 13]. Usually the glyoxalase system and related enzymes (e. g., aldo-ketoreductase) are sufficient to keep glycation reactions under control. However, in the aforementioned pathological conditions, dicarbonyl levels can surpass thresholds that are detrimental for the cell [14–17]. Furthermore, increased AGE formation has been implicated in biological aging [18]. It has been shown that in aging models like the nematode *Caenorhabditis elegans* and the filamentous fungus *Podospora anserina* a clear correlation between glycation stress and lifespan determination exists [19, 20].

*In vitro* glycation experiments using mostly MGO and a target molecule have been instrumental in elucidating the chemistry of the reactions that dicarbonyls can undergo [21–27]. In this study, we focused on two proteins which play an important role in the detoxification of the reactive oxygen species (ROS) superoxide (^.^O_2_^-^) in the mitochondrial intermembrane space (IMS): cytochrome-c and SOD1.

Cytochrome-c is a highly conserved α-helical protein with a size of 12 kDa that contains a covalently bound heme (type *c*) group. It is located in the IMS where it plays a vital role during cellular respiration by transferring electrons from coenzyme Q:cytochrome-c–oxidoreductase (complex III) to cytochrome-c oxidase (complex IV). Noteworthy, cytochrome-c is known as an important regulator of apoptosis [28, 29]. When mitochondria undergo MOMP (mitochondrial outer membrane permeabilization), cytochrome-c is released into the cytosol and activates caspase 9 via binding to Apaf-1 (apoptotic protease activating factor-1). Caspase 9 then activates the effector caspases 3 and 7, which are responsible for cellular disassembly. Furthermore, cytochrome-c emerged as a scavenger of superoxide anions without producing hydrogen peroxide, unlike superoxide dismutases (see below) [30]. In humans, cytochrome-c is encoded by the *CYCS* gene. Mutations in this gene are causally linked to thrombocytopenia. This disease is defined by the reduced number of platelets in circulating blood [31].

SOD1 is a dimeric protein of two identical 16 kDa subunits [32]. The protein contains β-strands and no α-helical structures. It is found in the cytosol [33], the nucleus [34] and, similar to cytochrome-c, in the IMS of mitochondria [33]. Each monomer contains a binuclear site containing one copper and one zinc ion. SOD1 catalyzes the disproportionation of two superoxide anions to yield the products hydrogen peroxide (H_2_O_2_) and molecular oxygen [32]. H_2_O_2_ can be subsequently degraded by catalase or peroxidases. Unsurprisingly, mutations in the gene encoding this important oxygen radical scavenger lead to severe pathologies. Amyotrophic lateral sclerosis 1 (ALS1) is linked to a number of mutations in the *SOD1* gene [35]. In ALS1, motor neurons in the brain and the brain stem are lost due to cell death. This condition ultimately results in fatal paralysis.

Here we show that MGO can robustly change several important structural properties of cytochrome-c and SOD1. In cytochrome-c, glycation influences the secondary structure by increasing the proportion of α-helices. Overall, the folding of this protein appears to be more compact. Cytochrome-c oligomers are formed under glycating conditions. Glycated SOD1 also shows a tendency to form oligomers whereas its secondary structure is unchanged. We find that AGE modifications lead to the formation of SOD1 oligomers. Although not directly demonstrated experimentally results from DSC experiments suggest that high MGO concentrations lead to de-metallation of the enzyme.

## Material and methods

### Sample preparation

Unless specified otherwise, 4 mg of horse heart cytochrome-c (Merck, cat. no. C2506) or SOD1 from bovine erythrocytes (Merck, cat. no. S7571) were dissolved in 2 ml 20 mM phosphate buffer (pH 7.0) and glycated with MGO (40% stock solution, Merck, cat. no. M0252) at different concentrations (10, 100, 1000 μM) at 25°C for 24 h. Samples were purified after the treatment using Amicon Ultra centrifugal filter units (Merck Millipore, Burlington, MA) according to the manufacturer’s instructions. Native (control) and glycated samples were obtained from the same stock solutions. Aliquots were collected and stored at -20°C overnight before use.

### SDS-PAGE and MG-H1 immunodetection

Sodium dodecyl sulfate (SDS) polyacrylamide gel electrophoresis (PAGE) was performed according to standard protocols to separate 30 μg aliquots of the samples. 4% stacking and 16% resolving gels were used. For immunodetection experiments proteins were transferred to Immun-Blot PVDF membranes (BIO-RAD cat. no. 162–0177) in transfer solution (10 mM Tris-base, 50 mM glycine, 10% methanol) for 1 h at 15 V using a ‘Semidry Electroblotter’ manufactured by Thermo Fisher Scientific (Model HEP-1). After a brief wash of the membrane in 1xTBS blocking of unspecific binding sites was performed for 1 h at room temperature in 1xTBS containing 3% skimmed milk powder. Subsequently, the membrane was probed using primary antibodies against the MGO modification 5-hydro-5-methylimidazolone (MG-H1) (Fig 1) (Oxiselect kit, part no. 281101, Cell Biolabs) for 16 h at 4°C. Detection was performed by using horseradish conjugated secondary antibodies (Oxiselect kit, part no. 230003, Cell Biolabs) for 1.5 h at room temperature and the ‘SuperSignal West Dura Extended Duration Substrate’ (Thermo Fisher Scientific, cat. no. 34075) according to the manufacturer's instructions. Signals were documented using the ‘Bio-Imaging System’ (MicroChemi, cat. no. 70-25-00).

### Differential scanning calorimetry

The samples (2 mg/ml) were degassed at 635 mm Hg, 500 rpm and room temperature for 5 min. Subsequently, they were loaded into the capillaries of a NanoDSC microcalorimeter (TA Instruments, USA) to record the heat capacity profiles. The spectra were obtained and analyzed using the software provided by the instrument manufacturer. Each experiment was performed twice using different samples prepared under the same conditions (heating rate 1°C/min).

### Fluorescence spectroscopy

Intrinsic fluorescence of cytochrome-c solutions was measured at 25°C with a FluoroMax-4 spectrofluorometer (HORIBA Jobin Yvon) equipped with a thermostatic cell chamber. The sample concentration was 0.1 mg/ml. The excitation wavelength was set to 280 nm. Emission fluorescence spectra were recorded between 300 and 450 nm setting the slit width to 10 nm and the temperature to 25°C. Experiments were repeated twice.

### Circular dichroism

To analyze the secondary structure of the proteins, circular dichroism (CD) spectra were recorded using a J-1100 spectropolarimeter (JASCO Inc., Easton MD, USA) with a 0.1 cm path length cell over the 190–240 nm range. The concentration of the samples was 0.2 mg/ml in 150 mM Tris-base, 50 mM NaCl buffer (pH 6.8). CD spectra of the native and glycated proteins were acquired at 50 nm/min. Each spectrum was obtained as an average of five scans to reduce noise and smoothed before structure analysis was made. The secondary structure prediction for cytochrome-c was performed using the CONTIN algorithm and SMP180 (optimized for 190–240 nm) reference sets accessed through the DICHROWEB online server [36]. For processing the structural raw data of SOD1, the SELCON3 algorithm was employed [37]. Experiments were repeated twice to confirm the reproducibility of the measurements.

## Results

### Analysis of protein modification and aggregation

**Fig 2:**
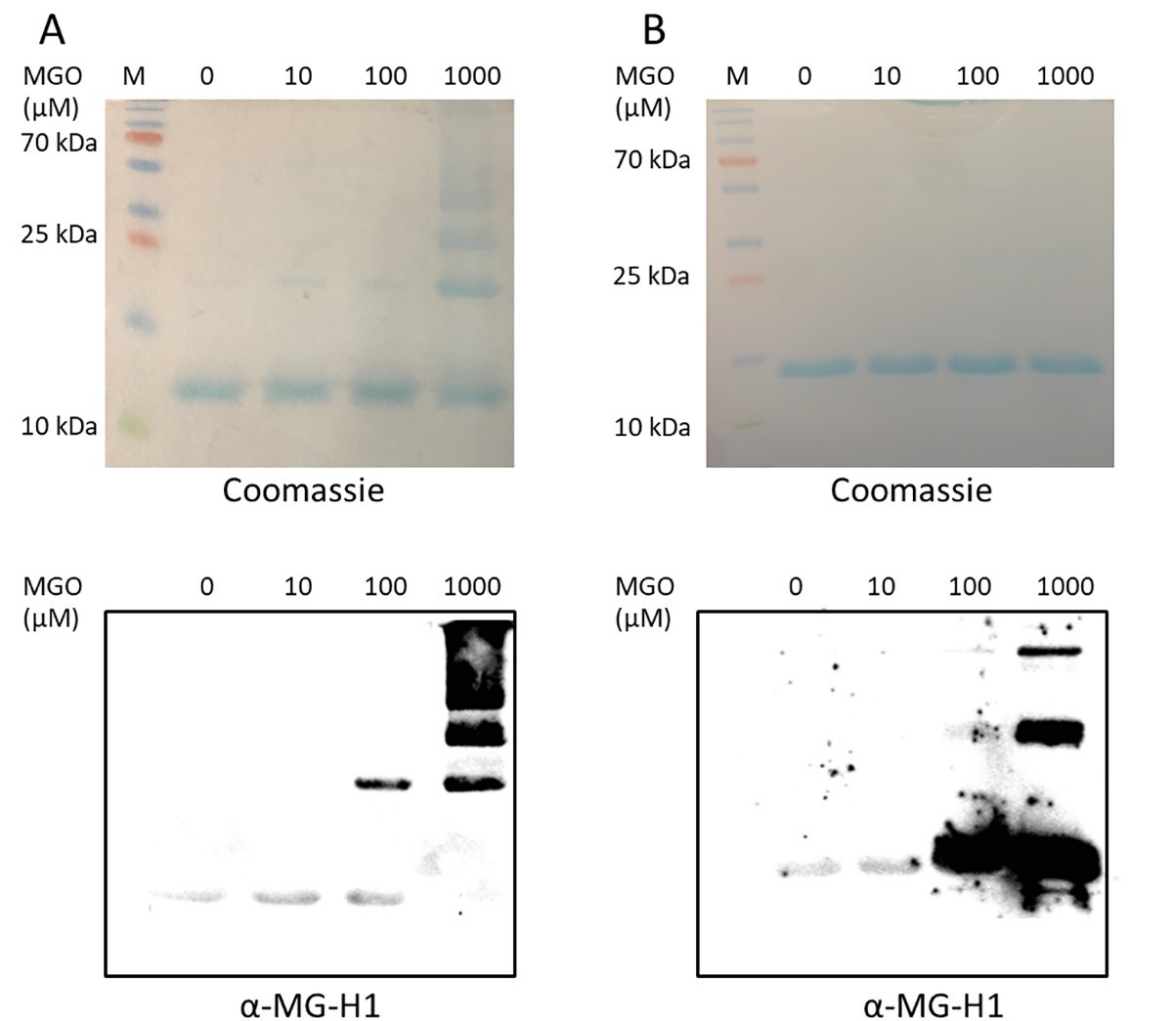
Analysis of MGO-mediated modification of cytochrome-c (A) and SOD1 (B). Untreated and MGO-subjected samples were resolved via SDS-PAGE and stained with Coomassie Blue (top) or α-MG-H1 antibodies after blotting (bottom). To reveal additional bands in the higher molecular weight range the SOD1 blot was overexposed. M: protein size standard. Representative results from two independent experiments are shown.

Both proteins, cytochrome-c and SOD1, were subjected to three different MGO concentrations (10 μM, 100 μM and 1 mM) overnight at 25°C. Although an MGO concentration of 1 mM does not necessarily reflect cellular dicarbonyl levels (see introduction), similar (and even higher) MGO levels are often used in the literature for studying the consequences of glycation on protein structure (e. g., [22–24, 38, 39]). Therefore, our choice facilitates the comparison of our data with these studies. After purification, samples from each treatment and untreated control were separated by means of SDS-PAGE (Fig 2A and 2B). Coomassie Blue staining was used to verify successful electrophoresis and revealed individual protein bands. Cytochrome-c is detected in all samples with an apparent molecular weight of 12 kDa (Fig 2A top). Treatment of cytochrome-c with the highest MGO concentration (1 mM) leads to the formation of several additional bands at approximately 24, 36 and 48 kDa (Fig 2A top), possibly by monomer-aggregation. To analyze the nature of these signals we decided to do an immunodetection experiment using primary antibodies that are specific for the detection of MG-H1. MG-H1 is an established marker of MGO-mediated protein modification (Fig 1) [40]. Weak signals originating from a band at 12 kDa are seen in the untreated control and the 10 μM and 100 μM MGO samples (Fig 2A bottom). Additionally, distinct bands are revealed at 24 kDa in the 100 μM and 1 mM MGO samples. Several bands of higher molecular weights are observed in the sample subjected to 1 mM MGO. These bands likely originate from cytochrome-c protein aggregation.

A similar approach was followed for analyzing the effects of MGO treatment on SOD1 (Fig 2B). Coomassie Blue staining revealed a single band with an apparent molecular weight of approximately 16 kDa (Fig 2B top). α-MG-H1 binding showed the presence of two additional bands with apparent molecular weights of approximately 32 and 64 kDa in the sample treated with 1 mM MGO (Fig 2B bottom). The 100 μM MGO sample does not include these bands but a very strong MG-H1 signal at approximately 16 kDa. By contrast, the untreated control and the 10 μM MGO sample show weakly decorated bands at approximately 16 kDa. Comparable to the situation with cytochrome-c, MGO concentrations at 100 μM and 1 mM are effectively modifying SOD1.

### Calorimetry response

**Fig 3:**
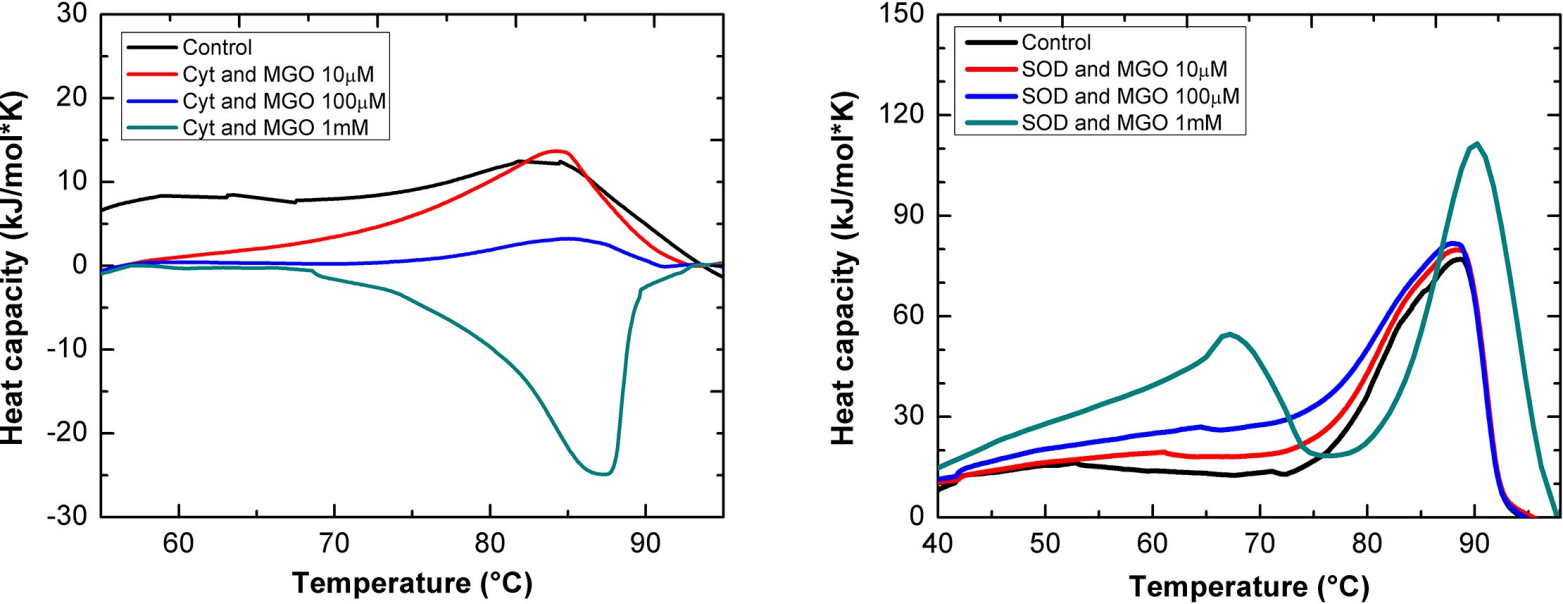
Differential calorimetry of cytochrome-c (A) and SOD1 (B) subjected to different concentrations of MGO. Calorimetry curves of native (i.e., unmodified) and MGO-modified horse heart cytochrome-c (2 mg/ml) and bovine SOD1 (2 mg/ml) in the temperature range 50–95°C, respectively.

Differential scanning calorimetry (DSC) monitoring protein unfolding was used to analyze whether MGO treatment leads to structural changes in the two proteins of interest. The behavior of cytochrome-c (native and treated with 10 μM and 100 μM MGO) reveals an endothermic state at approximately 83°C (Fig 3A). By contrast, there is a pronounced exothermic state of heat capacity observed in samples subjected to 1 mM MGO at approximately 87°C. In addition, a slight shift to the right of the calorimetric peak is observed as the concentration of MGO increases (from 83°C to 87°C for control and 1 mM MGO sample, respectively).

There are no significant differences in the calorimetry profile of SOD1 control and treatment with 10 μM and 100 μM MGO, respectively (Fig 3B). The curve peak is found around 88°C. However, when subjected to 1 mM MGO, SOD1 exhibits a different calorimetric profile that displays two peaks at approximately 66°C and 92°C. This might indicate the presence of two SOD1 populations (e. g., destabilized apoSOD1 not containing Cu and/or Zn and metallated SOD1).

### Fluorescence studies

**Fig 4:**
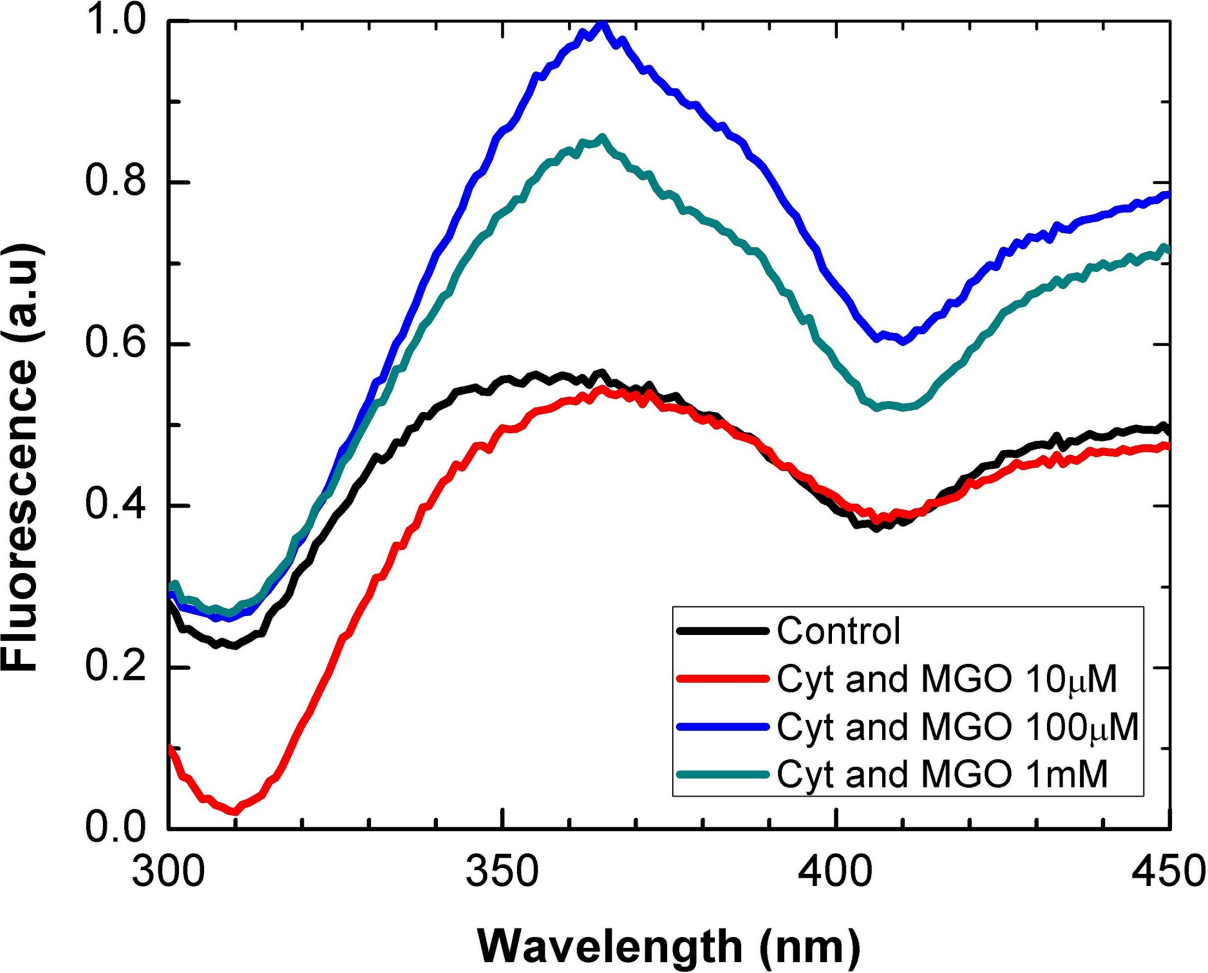
Fluorescence emission of cytochrome-c subjected to different concentrations of MGO. Fluorescence emission spectra of native (i.e., unmodified) and MGO-modified horse heart cytochrome-c (0.1 mg/ml) in the 300–450 nm region.

Fluorescence emission studies of tryptophan residues were conducted to test whether MGO treatment leads to alterations in the tertiary structure of cytochrome-c. These measurements were limited to cytochrome-c because SOD1 does not contain tryptophan. Spectra of intrinsic fluorescence of tryptophan reveal a pronounced emission increase in the samples glycated with 100 μM and 1 mM MGO, whereas cytochrome-c treated with 10 μM MGO shows a decrease in the wavelength range of 300–360 nm (Fig 4). These data suggest that the single tryptophan residue in cytochrome-c becomes less accessible to solvent contact by 100 μM and 1 mM MGO treatment which might be explained by monomer aggregation.

### Secondary structure studies

**Fig 5:**
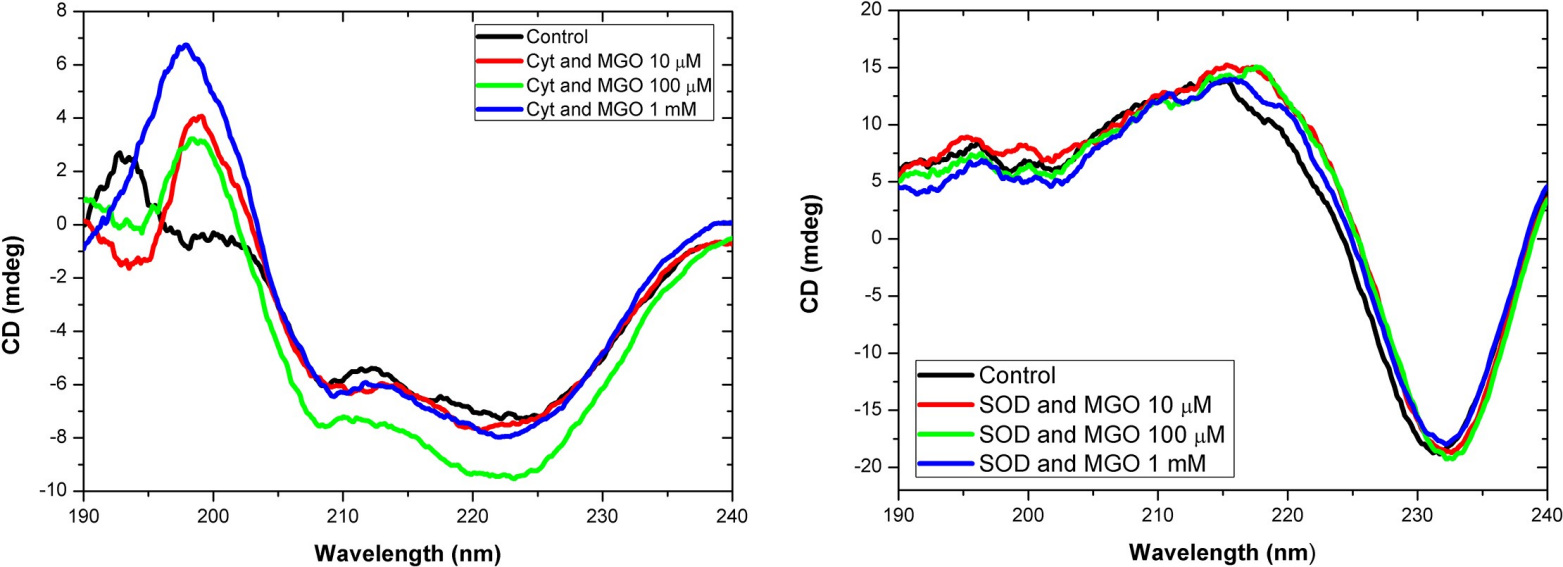
Circular dichroism of cytochrome-c (A) and SOD1 (B) subjected to different concentrations of MGO. CD spectra of native (i.e., unmodified) and MGO-modified horse heart cytochrome-c (0.2 mg/ml) and bovine SOD1 (0.2 mg/ml), respectively, in the far UV region (190–240 nm).

Analysis of far ultra violet CD (circular dichroism) spectra resulted in obtaining data on changes of the secondary structure observed in unglycated and glycated cytochrome-c (Fig 5A and Table 1). Notably, the occurrence of α-helical regions increases with MGO-mediated glycation (control: 0.476, 10 μM MGO: 0.500, 100 μM MGO: 0.603, 1 mM MGO: 0.609). Furthermore, unordered structures in cytochrome-c decrease with MGO levels at 100 μM and 1 mM (control: 0.344, 100 μM MGO: 0.323, 1 mM MGO: 0.234).

**Table 1:** Summary of CD data for cytochrome-c. The proportion of secondary structures was estimated from the deconvolution of CD spectra using the DICHROWEB online server with the CONTIN algorithm [36].

*Structural element*	*α-helix*	*β-strand*	*Turns*	*Unordered*
Cyt-c control	0.476	0.047	0.133	0.344
Cyt-c + 10 μM MGO	0.500	0.053	0.079	0.369
Cyt-c + 100 μM MGO	0.603	0.010	0.064	0.323
Cyt-c + 1 mM MGO	0.609	0.085	0.072	0.234

No structural changes were found after deconvolution of the CD data for the SOD1 samples (Fig 5B and Table 2). MGO seems not to influence the secondary structure of SOD1.

**Table 2:** Summary of CD data for SOD1. The proportion of secondary structures was estimated from the deconvolution of CD spectra using the DICHROWEB online server with the SELCON3 algorithm [37].

*Structural element*	*α-helix*	*β-strand*	*Turns*	*Unordered*
SOD1 control	0.000	0.293	0.219	0.329
SOD1 + MGO (all concentrations)	0.000	0.293	0.219	0.329

## Discussion

**Fig 6:**
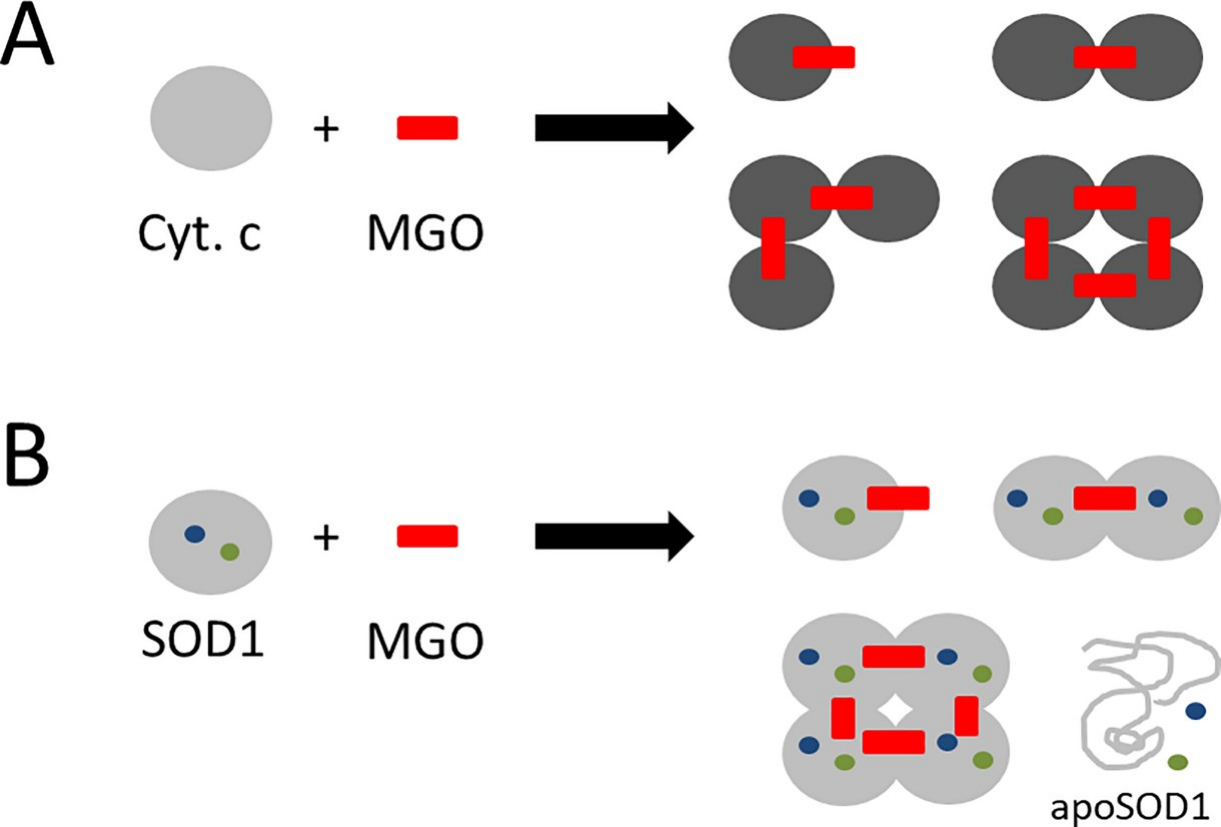
Structural effects of MGO treatment on cytochrome-c (A) and SOD1 (B). **(A)** Cytochrome-c monomers aggregate in the presence of sufficiently high MGO doses. Dimers, trimers and tetramers were revealed by SDS-PAGE and MG-H1 immunodetection. A darker shade indicates more compact folding (as observed by DSC, CD and fluorescence measurements). **(B)** MGO treatment of SOD1 also leads to monomer aggregation (dimers and tetramers were electrophoretically resolved). DSC measurements reveal a protein population exhibiting a significantly reduced melting temperature. This could indicate the presence of apoSOD1 (de-metallated form of the enzyme).

Our aim was to study effects of the reactive dicarbonyl MGO on structural properties of cytochrome-c and SOD1. The immunodetection analysis using antibodies directed against the MG-H1 modification reveals that at an MGO concentration of 100 μM an additional signal with twice the apparent molecular weight of the cytochrome-c monomer appears. Further bands at three- and four-fold the size of the cytochrome-c monomer are revealed at a MGO concentration of 1 mM. These findings are in line with a previous work that reported the aggregation of cytochrome-c oligomers through glycation [22]. The aggregates are suggested to stabilize glycated and partially unfolded monomers. Oligomers composed of up to four units are described in such earlier study [22]. Our results support this finding, as we were able to detect dimers, trimers and tetramers (Fig 6). Furthermore, the fact that we could detect the glycation modification with an antibody directed against MG-H1 supports the earlier finding that cytochrome-c is indeed MG-H1-modified (at arginine 92) [22].

In our study, MGO concentrations of 100 μM and 1 mM are effectively glycating SOD1. At the latter MGO concentration, SOD1 oligomerization is observed, confirming an earlier result by the Iannuzzi lab [24] (Fig 6).

Taken together, in both cytochrome-c and SOD1 glycation leads to protein aggregation/oligomer stabilization. Interestingly, for SOD1 higher MGO levels are required to observe this effect (1 mM MGO vs. 100 μM MGO). Probably the modification sites in cytochrome-c are more accessible to the solvent and thus MGO.

DSC allows the determination of heat capacity of a solution (expressed in kJ/mol K). This sensitive technique can robustly reveal the unfolding behavior of proteins. Furthermore, changes of the oligomeric state of proteins can be analyzed. Unglycated and low-level glycated cytochrome-c from horse heart shows a maximum of heat capacity at approximately 83°C which is in accordance with the value reported in Privalov 1979 [41] (the values are not identical because different pH conditions were used in this study among other experimental variations). This endothermic state is probably due to thermal denaturation of the protein. In the temperature range between 50 and 80°C a reduced heat capacity of the protein treated with 10 μM MGO is revealed. Remarkably, the calorimetric behavior of cytochrome-c is drastically altered after being glycated with 1 mM MGO: a pronounced decrease of heat capacity is observed, with a minimum at approximately 87°C. This effect could be due to aggregation or precipitation of a high number of cytochrome-c oligomers. As glycation was shown to induce oligomerization of cytochrome c (Fig 2A and [22]) this change might represent the exothermic association of (partially) unfolded cytochrome-c monomers to oligomers. Treatment with 100 μM MGO stabilizes cytochrome-c which correlates with a temperature increase of the thermal denaturation peak (shift to the right in a calorimetry diagram [42]).

SOD1 that is subjected to a gradual heat increase reveals a pronounced augmentation of heat capacity at approximately 85°C. Glycation using 10 μM and 100 μM MGO has no significant effect on the denaturation behavior. In contrast, glycation with a high level of MGO (1 mM) leads to the appearance of an additional maximum at approximately 66°C and a slight shift of the main maximum to 90°C. The former peak might be based on a de-metallated form of the protein (i. e., apoSOD1) that is present in the sample whereas the latter indicates a slightly stabilizing effect of MGO on SOD1 which was reported previously [24] and which we could demonstrate by immunodetection (Fig 2B). The pronounced aggregation of SOD1 shown in the immunodetection experiments strengthens our assumption that apoSOD1 is present in the sample treated with 1 mM MGO because it was shown that apoSOD1 has a much stronger tendency to form oligomers compared to metallated SOD1 [24]. Furthermore, chemical modifications that increase the surface hydrophobicity of a water soluble protein like SOD1 could generally lead to lower melting temperatures, but there are noted exceptions such as in the acetylation of lysine in α-amylase (which increases the melting temperature of α-amylase according to DSC [43]). These exceptions are rationalized in terms of optimizing the surface packing of residues in proteins with high amounts of surface “grooves”. This might explain the increased Tm transition of (metallated) SOD1 upon incubation in 1 mM MGO. Furthermore, it should be taken into account that glycation can change the charge of a protein (Fig 1). Therefore it is plausible that glycation might have electrostatic effects on protein structure similar to acetylation [44, 45].

In this study fluorescence measurements were conducted to test whether there are changes in cytochrome-c conformation (tertiary structure) upon glycation with MGO. In comparison with untreated cytochrome-c, incubation with 10 μM MGO leads to a decrease of fluorescence in the wavelength range of 300 to 360 nm. A drop of fluorescence could be due to exposure of tryptophan to the aqueous medium. However, when higher concentrations of MGO are used, fluorescence is markedly increased compared to the untreated control. This might indicate a more compact folding (indication of “buried” tryptophan residues) of cytochrome-c or aggregation, thereby preventing exposure of tryptophan to the surrounding medium and subsequent quenching of fluorescence emission. Cytochrome-c folding has been described as a complex process that comprises both slow and fast folding steps which are highly influenced by the pH of the buffer [46, 47]. Therefore, it is conceivable that MGO-mediated modification of the basic amino acid residues arginine and lysine could lead to an altered folding process.

The analysis of circular dichroism of a protein sample is a valuable tool to probe its secondary structure [48]. Interestingly, the content of alpha helical structural features in cytochrome-c increases with the application of rising MGO concentrations, whereas the proportion of unordered regions decreases. These findings are in contrast to a previous study that described a loss of alpha helices and an increase of unordered and beta-strand features upon glycation of cytochrome-c [39]. Reasons for these differences might be found in different glycation protocols (e. g., 37°C vs 25°C incubation temperature). Our data suggest that MGO-modification leads to a more compact folding of cytochrome-c, which might explain its structural changes observed in the calorimetry (Fig 3A) and fluorescence (Fig 4) measurements.

We could not observe changes of beta strand-structured regions in SOD1 with increasing MGO concentrations. Previously it was reported that treatment of human SOD1 leads to a gradual decrease of beta strands with time [24]. Similar to the cytochrome-c measurements, the differences to our study might be due to experimental differences in the glycation protocol (37°C vs 25°C incubation temperature, up to 1 mM MGO instead of 5 mM MGO and 24 h instead of at least 48 h of incubation).

Currently it is unknown whether the structural changes we have observed in cytochrome-c and SOD1 have an impact on their enzymatic activity. Furthermore, release of metal ions from SOD1 might increase formation of hydroxyl radicals by Fenton chemistry [49], thereby increasing the oxidative stress in the cell. Cytochrome-c oligomerization could lead to an impaired “shuttling” of electrons from complex III to complex IV in the respiratory chain of mitochondria. Impeded electron transport in the respiratory chain has been implied in elevated levels of reactive oxygen species [50]. Further studies might yield interesting insights into the interplay of glycation of cytochrome-c and SOD1 and the formation of reactive oxygen species.

## Conclusions

Several structural parameters of proteins are influenced by MGO treatment. Specifically, we found that glycation of cytochrome-c from horse heart leads to monomer aggregation, an altered secondary structure (increase in α-helical content), more compact folding and altered thermal unfolding behavior. Subjecting SOD1 to MGO results in subunit aggregation and dimer/tetramer formation. Our results also suggest de-metallation of SOD1.

## Supporting information

S1 Raw Images (PDF)
