# Supplementary material for: Analyzing structural alterations of mitochondrial intermembrane space superoxide scavengers cytochrome-c and SOD1 after methylglyoxal treatment
Source: PLoS One. 2020 Apr 30;15(4):e0232408. doi: 10.1371/journal.pone.0232408 (PMC7192434; doi:10.1371/journal.pone.0232408)
Supplement: S1 Raw Images — (PDF) [file pone.0232408.s001.pdf]

2 A top

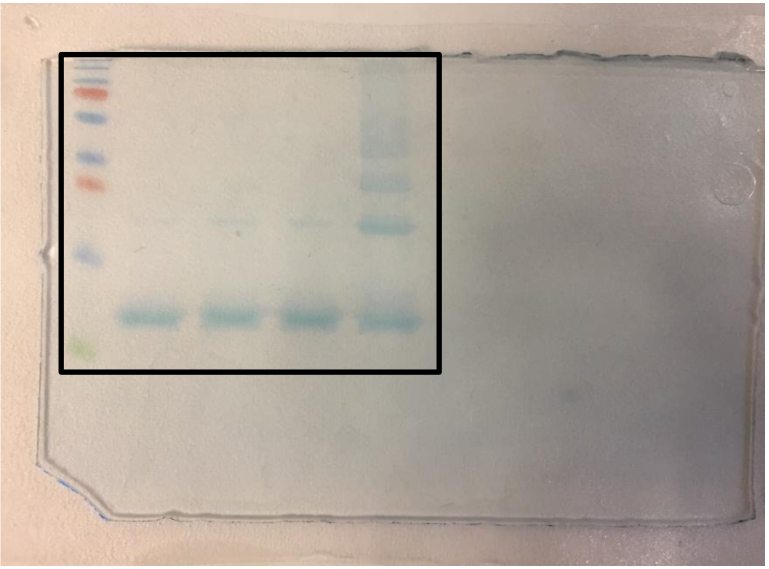

Coomassie Blue, captured with a digital camera (Olympus)

Mercado-Uribe et al.

2 A bottom

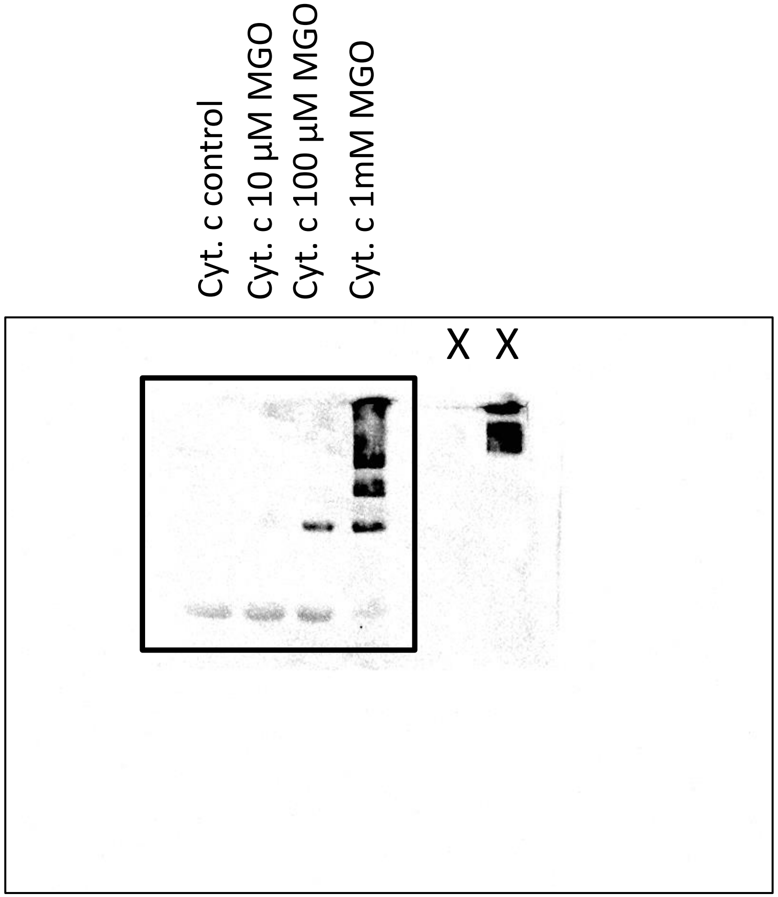

α-MG-H1, captured with MicroChemi 'Bio-Imaging System'

Mercado-Uribe et al.

2 B top

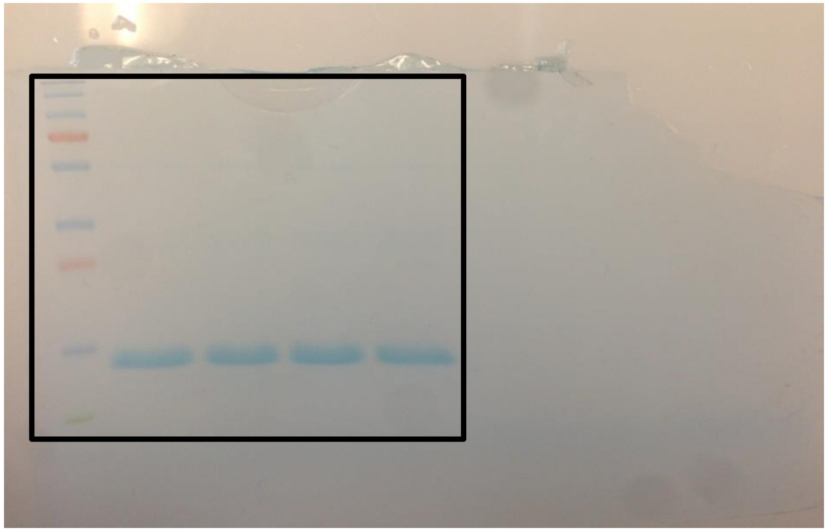

Coomassie Blue, captured with a digital camera (Olympus)

Mercado-Uribe et al.

2 B bottom

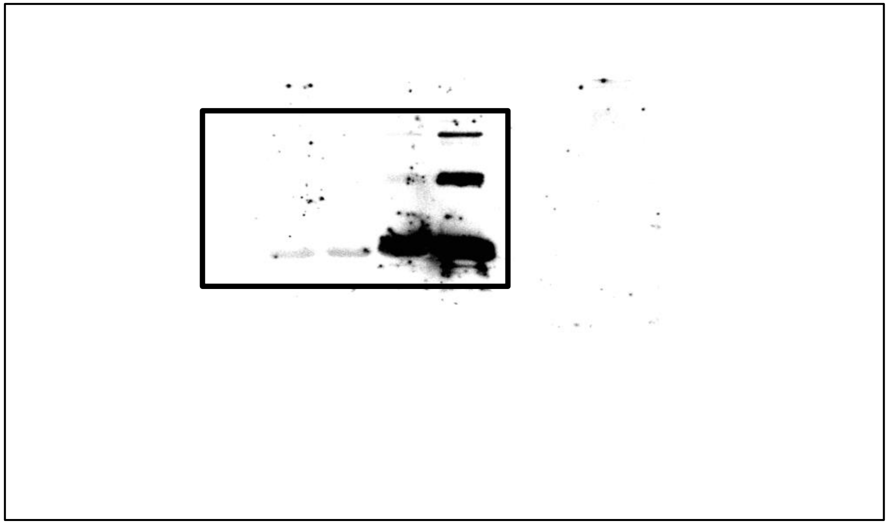

α-MG-H1, captured with MicroChemi 'Bio-Imaging System'

Mercado-Uribe et al.
